# Nitrogen-fixing Ability and Nitrogen Fixation-related Genes of Thermophilic Fermentative Bacteria in the Genus *Caldicellulosiruptor*

**DOI:** 10.1264/jsme2.ME21018

**Published:** 2021-06-10

**Authors:** Yuxin Chen, Arisa Nishihara, Shin Haruta

**Affiliations:** 1 Department of Biological Sciences, Tokyo Metropolitan University, 1–1 Minami-Osawa, Hachioji, Tokyo 192–0397, Japan; 2 Bioproduction Research Institute, National Institute of Advanced Industrial Science and Technology (AIST), Tsukuba, Ibaraki 305–0856, Japan

**Keywords:** diazotroph, hot spring, thermophile, fermentation, *Firmicutes*

## Abstract

Fermentative nitrogen-fixing bacteria have not yet been examined in detail in thermal environments. In the present study, we isolated the thermophilic fermentative bacterium, strain YA01 from a hot spring. This strain grew at temperatures up to 78°C. A phylogenetic analysis based on its 16S rRNA gene sequence indicated that strain YA01 belonged to the genus *Caldicellulosiruptor*, which are fermentative bacteria in the phylum *Firmicutes*, with 97.7–98.0% sequence identity to its closest relatives. Strain YA01 clearly exhibited N_2_-dependent growth at 70°C. We also confirmed N_2_-dependent growth in the relatives of strain YA01, *Caldicellulosiruptor hydrothermalis* 108 and *Caldicellulosiruptor kronotskyensis* 2002. The nitrogenase activities of these three strains were examined using the acetylene reduction assay. Similar activities were detected for all tested strains, and were slightly suppressed by the addition of ammonium. A genome analysis revealed that strain YA01, as well as other *Caldicellulosiruptor*, possessed a gene set for nitrogen fixation, but lacked the *nifN* gene, which encodes a nitrogenase iron-molybdenum cofactor biosynthesis protein that is commonly detected in nitrogen-fixing bacteria. The amino acid sequences of nitrogenase encoded by *nifH*, *nifD*, and *nifK* shared 92–98% similarity in *Caldicellulosiruptor*. A phylogenetic tree of concatenated NifHDK sequences showed that NifHDK of *Caldicellulosiruptor* was in the deepest clade. To the best of our knowledge, this is the first study to demonstrate the nitrogen-fixing ability of fermentative bacteria at 70°C. *Caldicellulosiruptor* may have retained an ancient nitrogen-fixing enzyme system.

Nitrogen is one of the most abundant and important elements for life. Nitrogen-fixing microorganisms play significant roles in converting atmospheric N_2_ gas to ammonia in ecosystems. According to a review by Postgate (1998), the first nitrogen-fixing bacteria or diazotrophs were discovered by Winogradsky in 1893. Nitrogen-fixing microorganisms have been reported in 16 phyla in *Bacteria* and 1 phylum in *Archaea* from various environments (Mus *et al.*, 2019). Aerobic free living and symbiotic Proteobacteria and phototrophs have been widely reported (Martinez-Romero, 2006; Flores *et al.*, 2015; Wasai and Minamisawa, 2018), and the nitrogen-fixing ability of anaerobic respiratory bacteria, such as *Anaeromyxobacter* in soil, has recently been attracting increasing attention (Masuda *et al.*, 2020). In 1988, fermentative nitrogen-fixing bacteria were reported in the genus *Clostridium* in *Firmicutes* (Leschine *et al.*, 1988); however, limited information is currently available on fermentative nitrogen-fixing bacteria. Nitrogen fixation by fermentative metabolism utilizing polysaccharides (*e.g*., cellulose) has been suggested to play an important role in nitrogen cycles in soil and animal intestines (Monserrate *et al.*, 2001; Yamada *et al.*, 2007).

Nitrogen fixation is achieved by multiple proteins encoded by *nif* genes (Raymond *et al.*, 2004). Phylogenetic examinations indicated that nitrogenase genes originated in archaea and were horizontally transferred to bacteria (Boyd *et al.*, 2011a). The *nifH* gene encoding the nitrogenase reductase subunit of nitrogenase is widely regarded as an indicator of the existence of diazotrophs (Zehr *et al.*, 2003). The diversity and distribution of *nifH* genes have been analyzed in natural ecosystems, including thermal environments (Mehta *et al.*, 2003; Hamilton *et al.*, 2011; Zehr, 2011; König *et al.*, 2016; Pajares and Bohannan, 2016; Nishihara *et al.*, 2018c). The nitrogen-fixing methanogenic archaeon, *Methanocaldococcus* FS406-22, was isolated from a deep-sea hyperthermal vent and its nitrogen-fixing ability was demonstrated at temperatures up to 92°C (Mehta and Baross, 2006). In 1986, nitrogen-fixing ability was reported in a thermophilic cellulose-degrading fermentative bacterium that grew at 60°C (Bogdahn and Kleiner, 1986a). Nishihara *et al.* (2018b) recently reported the nitrogen-fixing ability of H_2_-oxidizing aerobic bacteria in the genus *Hydrogenobacter* sp. in the deeply branching phylum *Aquificae* at 70°C; this is the highest temperature observed for N_2_ fixation in *Bacteria*. However, thermophilic isolates that grow at temperatures higher than 70°C are still limited.

In Nakabusa Hot Spring (Nagano, Japan), a sulfidic and slightly alkaline hot spring, chemosynthetic microbial communities develop well at temperatures higher than 70°C (Nakagawa and Fukui, 2002, 2003; Kimura *et al.*, 2010; Nishihara *et al.*, 2018a), and these communities are dominated by H_2_-/sulfur-oxidizing bacteria in *Aquificae* (Tamazawa *et al.*, 2012, 2016). The nitrogenase activity of the communities was detected *ex situ* at 70°C under anaerobic conditions (Nishihara *et al.*, 2018a). Nishihara *et al.* (2018c) also performed a *nifH* gene amplicon analysis of chemosynthetic microbial communities at temperatures between 72 and 77°C, and the findings obtained showed that the relative abundance of the *nifH* gene from *Caldicellulosiruptor* were 7.42, 48.97, 73.12, and 94.58% in the four samples analyzed. The genus *Caldicellulosiruptor* comprises thermophilic fermentative bacteria that exhibit cellulolytic activities (Zverlov *et al.*, 1998; Blumer-Schuette *et al.*, 2012; Brunecky *et al.*, 2013) and is widely distributed in globally diverse thermal environments (Lee *et al.*, 2018; Blumer-Schuette, 2020). Genes related to nitrogen fixation are found in the genomes of some species in this genus (CP002330.1, CP002326.1, CP002219.1, CP003001.1, CP000679.1, LACO01000001.1, LACM01000001.1, and LACN01000001.1); however, their nitrogenase activities and dinitrogen-dependent growth have not yet been demonstrated.

In the present study, we isolated thermophilic fermentative bacteria using a combined nitrogen-poor medium from microbial communities developed at approximately 80°C in Nakabusa Hot Spring and characterized their nitrogen-fixing abilities and genetic features in comparisons with their closest relatives.

## Materials and Methods

### Isolation of bacteria under nitrogen-fixing conditions

Pale tan-colored microbial mats developed in hot spring water at 78.3°C were collected at Nakabusa Hot Springs (36° 23′ 20″ N 137° 44′ 22″ E), Nagano, Japan on January 8th, 2018. Hot spring water was slightly alkaline (pH 8.5–8.9) and contained 5.0–6.1‍ ‍μmol L^–1^ of ammonia (Kato *et al.*, 2004), but not nitrate or nitrite (Kato *et al.*, 2004; Kimura *et al.*, 2010). Samples were immediately injected into the anoxic medium in glass vials (see below) with attempts to avoid oxygen contamination at the sampling site. The vials were stored in hot spring water at 60–75°C for 7 h during transportation to our laboratory and then incubated at 70°C.

Winogradsky’s nitrogen-poor mineral medium (Tchan and New, 1984) was prepared with a slight modification and used for the cultivation and isolation of bacteria (L^–1^): 0.28‍ ‍g K_2_HPO_4_, 0.053‍ ‍g KH_2_PO_4_, 0.12‍ ‍g MgSO_4_·7H_2_O, 0.125‍ ‍g NaCl, 0.05‍ ‍g yeast extract, 0.01‍ ‍g CaCl_2_·2H_2_O, 2.5‍ ‍mg FeSO_4_·7H_2_O, 2.5‍ ‍mg MnSO_4_·5H_2_O, 2.5‍ ‍mg Na_2_MoO_4_·2H_2_O, 2.5‍ ‍g glucose, 2.5‍ ‍g sucrose, and 2.5‍ ‍g Na-pyruvate. The pH of the medium was adjusted to 7.5. Twenty milliliters of the medium was placed into a 70-mL glass vial. The vial was sealed with a butyl rubber stopper and aluminum cap, and then autoclaved after the gas phase had been replaced with N_2_. In total, 0.5‍ ‍mL of the culture was repetitively sub-cultured every week in fresh medium. After 10 sub-cultivations, an isolate was obtained by the twice dilution-to-extinction technique. The single morphology of microbial cells was confirmed under a phase-contrast microscope (Axio Imager 2; Carl Zeiss).

### Cultivation and maintenance of bacteria

*Caldicellulosiruptor hydrothermalis* 108, *Caldicellulosiruptor bescii* DSM 6725, and *Caldicellulosiruptor kronotskyensis* 2002 were obtained from DSMZ (Germany) (Miroshnichenko *et al.*, 2008; Yang *et al.*, 2010). Bacterial strains were cultivated at 70°C under the N_2_:CO_2_ (8:2) gas phase in medium containing the following (L^–1^): 0.068‍ ‍g KH_2_PO_4_, 0.087‍ ‍g K_2_HPO_4_, 2.09‍ ‍g MOPS, 0.33‍ ‍g KCl, 0.25‍ ‍g NH_4_Cl, 0.6‍ ‍g MgSO_4_·7H_2_O, 0.4‍ ‍g NaCl, 0.1‍ ‍g CaCl_2_·2H_2_O, and 10‍ ‍mL trace minerals. Twenty milliliters of the medium was prepared in 50-mL glass vials sealed with a butyl rubber stopper and aluminum cap. After autoclaving, 0.8‍ ‍mL of a filter-sterilized 10% cellobiose solution, 0.2‍ ‍mL of a 5% NaHCO_3_ solution, and 0.2‍ ‍mL of a vitamin solution were injected into the vials. The trace minerals solution comprised the following (L^–1^): 1.5‍ ‍g nitrilotriacetic acid, 3.0‍ ‍g MgSO_4_·7H_2_O, 0.5‍ ‍g MnSO_4_·5H_2_O, 1.0‍ ‍g NaCl, 0.1‍ ‍g FeSO_4_·7H_2_O, 0.1‍ ‍g CaCl_2_·2H_2_O, 0.1‍ ‍g CoCl_2_·6H_2_O, 0.13‍ ‍g ZnCl_2_, 0.01‍ ‍g CuSO_4_, 0.01‍ ‍g AlK(SO_4_)·12H_2_O, 0.01‍ ‍g H_3_BO_3_, 0.025‍ ‍g Na_2_MoO_4_·2H_2_O, 0.024‍ ‍g NiCl_2_·6H_2_O, and 0.025‍ ‍g Na_2_WO_4_·H_2_O. The components of the vitamin solution were as follows (L^–1^): 2‍ ‍mg biotin, 2‍ ‍mg folic acid, 10‍ ‍mg pyridoxine HCl, 5‍ ‍mg riboflavin, 5‍ ‍mg thiamine, 5‍ ‍mg nicotinic acid, 5‍ ‍mg pantothenic acid, 0.1‍ ‍mg vitamin B_12_, *p*-aminobenzoic acid, and 5‍ ‍mg thioctic acid.

### 16S rRNA gene sequence analysis

Bacterial cells were collected by centrifugation and total DNA was extracted according to a method reported by Noll *et al.* (2005). A DNA fragment of the 16S rRNA gene was PCR-amplified using the 27F and 1492R primers (Lane *et al.*, 1985; Lane, 1991), and amplified DNA after purification by the LaboPass PCR purification Kit (CosmoGenetech) was directly sequenced using BigDye terminator kit v3.1 on an ABI3130 Genetic Analyzer (Applied Biosystems). Sequences were compared using the BLAST program (Altschul *et al.*, 1997) with those available in the DDBJ/EMBL/GenBank databases.

### Genome analysis

Total DNA was extracted from bacterial cells by Qiagen Genomic-tip 100/G for bacterial cells (Qiagen) and sequenced by Bioengineering Lab. using the combination of DNBSEQ-G400 (MGI Tech) and GridION with the flow cell-type R9.4.1 (Oxford Nanopore Technologies) platform. Regarding DNBSEQ-G400, DNA was fragmented using a Covaris S2 ultrasonicator (Covaris) to obtain 500-bp DNA fragments. The DNBseq DNA library was prepared according to the manufacturer’s instructions and sequenced using DNBSEQ-G400 (pair-end 150-bp reads). In GridION, the library was prepared using the Ligation Sequence Kit (SQK-LSK109) after barcoding using Native Barcoding Expansion (Oxford Nanopore Technologies EXP-NBD104) and was then sequenced. In the DNBSEQ-G400 analysis, 3,500,000 read pairs (1.05 Gbp) were sampled using Seqkit (v. 0.11.0) (Shen *et al.*, 2016) and quality filtered using Sickle (v. 1.2.3) (Joshi and Fass, 2011) with the parameters -q 20 -l 127. In the GridION analysis, adaptors of the reads obtained were trimmed using Porechop (v. 0.2.3) (Wick *et al.*, 2017) and quality filtered using Filtlong (v. 0.2.0) (https://github.com/rrwick/Filtlong) with the parameters --‍min_length 1000 --target_bases 250000000, and processed error-prone reads using Canu v1.8 (Koren *et al.*, 2017). A total of 3,033,015 reads (DNBSEQ) and 67,859 reads (GridION) were obtained after quality filtering and subjected to use for hybrid assembly by Unicycler (v. 0.4.7) (Phillippy *et al.*, 2017) with the default setting. The assembled genome was annotated using Prokka v1.14.0 (Seemann, 2014). Proteins involved in nitrogen fixation were visualized using ‘gggenes’ (https://CRAN.R-project.org/package=gggenes) with ‘ggplot2’ in R package (R Foundation, Vienna, Austria) (https://www.R-project.org/) (Seemann, 2014; Wickham, 2016).

### Nitrogen fixation gene cluster and phylogenetic analysis

A concatenated phylogenetic tree of Nif/Anf/VnfHDK was constructed using 276 Nif/Anf/VnfHDK protein homologs, which were located in operons in 235 genomes including the genome newly analyzed in the present study. Genomes harboring Nif/Anf/VnfHDK homologues were examined using AnnoTree v1.2 (Boyd *et al.*, 2011b; Mendler *et al.*, 2019; Garcia *et al.*, 2020), collected from the National Center for Biotechnology Information database, and annotated using Prokka v1.14.0 (Seemann, 2014). Amino acid sequences were aligned using Mafft v7.427 (Katoh *et al.*, 2002). Maximum likelihood trees were constructed using RAxML-NG v. 0.9.0 with the LG+F+G4 model and 100 bootstrap replicates (Kozlov *et al.*, 2019). Bootstrap support values were recalculated by BOOSTER (v0.1.2) (Lemoine *et al.*, 2018). The MarHDK protein in *Rhodospirillum rubrum* ATCC11170 (WP_011388553.1, WP_011388552.1, and WP_011388551.1) was used as the outgroup (North *et al.*, 2020).

### Growth capability in nitrogen-poor media

Modified Winogradsky’s nitrogen-poor mineral medium (described above) was used to assess N_2_-dependent growth. Ten milliliters of medium was prepared in 32-mL glass test tubes sealed with butyl rubber stoppers and screw caps and the gas phase of the culture tube was filled with N_2_ or argon (Ar) gas. In total, 0.5‍ ‍mL of bacterial cultures pre-cultivated in nitrogen-poor medium were inoculated into fresh nitrogen-poor medium. To test the growth capability of *C. bescii* DSM 6725, a pre-cultivation was conducted using medium supplemented with 2‍ ‍mmol L^–1^ of NH_4_Cl. Growth in the culture was assessed by measurements of optical density (OD) at 660‍ ‍nm (miniphoto 518R; Taitec). Cultivation medium containing 2‍ ‍mmol L^–1^ NH_4_Cl was also used to compare N_2_-dependent growth with growth on ammonium.

### Nitrogenase activity by the acetylene reduction assay

Nitrogenase activity was detected using the acetylene reduction assay method (Leschine *et al.*, 1988). In total, 0.5‍ ‍mL of bacterial pre-cultures in modified Winogradsky’s nitrogen-poor mineral medium was inoculated into 10‍ ‍mL of the same medium in 25-mL glass vials and cultivated under a N_2_ gas atmosphere. At the exponential growth phase, a portion (0.5‍ ‍mL) of the culture solution was removed and mixed with 0.05‍ ‍mL of 10% Formalin Neutral Buffer Solution (pH 7.4–7.5, Fujifilm Wako Pure Chemical) to fix cells for the cell number count. The gas phase of culture vials was then replaced with N_2_ gas and 1.5‍ ‍mL of 99.9999% acetylene gas was injected into each vial. Vials were incubated at 70°C and, after a 24-h incubation, 1‍ ‍mL of 37% neutralized formaldehyde was added to stop the reaction. The production of ethylene by the reduction of acetylene was quantified using a GC-2014 gas chromatograph equipped with a flame ionization detector (Shimadzu) and 80/100 Porapak T (GL Science) column. Analysis conditions were as follows; carrier gas, N_2_ gas; column temperature, 70°C; injection temperature, 100°C; detector temperature, 100°C. Fresh medium containing no bacterial cells was prepared in the vial as a negative control to confirm abiotic ethylene production under the same conditions.

### Nucleotide sequence accession number

The 16S rRNA gene sequence was deposited in the DDBJ/EMBL/GenBank databases with the accession number LC603168. The accession numbers of the genomic sequences of strain YA01 were AP024480 (chromosome) and AP024481 and AP024482 (two plasmids).

## Results

### Bacterial isolate from the hot spring under anaerobic nitrogen-fixing conditions

Pale tan-colored microbial mats collected at 78.3°C from Nakabusa Hot Spring were directly inoculated into glass vials with modified Winogradsky’s nitrogen-poor mineral medium and anaerobically incubated at 70°C. After several sub-cultivations at one-week intervals, a stable enrichment culture was obtained. A pure culture containing cells of a single morphotype, *i.e.*, short rods (Fig. S1), was obtained by dilution-to-extinction and the isolate was designated as strain YA01. Strain YA01 grew at temperatures up to 78°C. The 16S rRNA gene sequence of strain YA01 (1,474 bp) showed 98.0, 97.7, and 97.7% identities to those of its closest relatives, *C. hydrothermalis* 108, *C. bescii* DSM 6725, and *C. kronotskyensis* 2002, respectively. This result indicated that strain YA01 was a species of the genus *Caldicellulosiruptor*.

### Nitrogen-fixation related genes in *Caldicellulosiruptor*

DNBSEQ-G400 and GridION runs resulted in the generation of approximately 12,782,840 reads with a total of 1,917‍ ‍Mbp and 155,919 reads with a total of 255‍ ‍Mbp, respectively. The complete genome of strain YA01 consisted of a single chromosome with a length of 2,592,764 bp and two plasmids with lengths of 3,514 and 1,547 bp. The G+C content of the genome was 34.8%. Coding potential predictions identified 2,412 protein-coding genes, three rRNA operons, and 47 tRNA genes. Three 16S rRNA genes had the same sequence, which was identical to the 16S rRNA gene sequence amplified by PCR. Average nucleotide identity (ANI) between strain YA01 and its close relatives (*C. hydrothermalis* 108, *C. bescii* DSM 6725, and *C. kronotskyensis* 2002) calculated using an ANI calculator (http://enve-omics.ce.gatech.edu/ani/) (Rodriguez-R, L.M., and Konstantinidis, K.T. 2016 The enveomics collection: a toolbox for specialized analyses of microbial genomes and metagenomes. *PeerJ Preprints* 4: e1900v1) ranged between 90.31 and 91.10%.

**Fig. 1.**
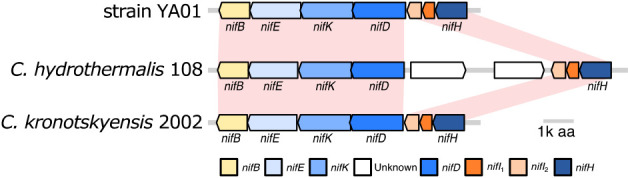
Nitrogen fixation gene clusters for strain YA01 and its closest relatives, *Caldicellulosiruptor hydrothermalis* 108 and *Caldicellulosiruptor kronotskyensis* 2002. The arrow indicates the transcriptional direction.

Annotation results showed that the chromosome of strain YA01 contained at least seven proteins involved in nitrogen fixation: three genes for nitrogenase structural proteins (NifH, NifD, and NifK), two proteins involved in the biosynthesis of MoFe protein cofactors (NifB and NifE), and two proteins of the PII family involved in posttranslational nitrogenase regulation (NifI_1_ and NifI_2_) (Arcondéguy *et al.*, 2001; Dodsworth *et al.*, 2005; Burén *et al.*, 2020) (Fig. 1). These seven genes were also identified in eight out of the 14 genomes in the genus *Caldicellulosiruptor* available in GenBank; *C. morganii* Rt8.B8 (accession no. LACO01000001.1), *C. naganoensis* NA10 (accession no. LACN01000001.1), *C. danielii* strain Wai35.B1 (accession no. LACM01000001.1), *C. lactoaceticus* 6A (accession no. CP003001.1), *C. kronotskyensis* 2002 (accession no. CP002330.1), *C. kristjanssonii* I77R1B (accession no. CP002326.1), *C. hydrothermalis* 108 (accession no. CP002219.1), and *C. saccharolyticus* DSM 8903 (accession no. CP000679.1) (Van De Werken *et al.*, 2008; Kataeva *et al.*, 2009; Blumer-Schuette *et al.*, 2011; Wai *et al.*, 2015; Blumer-Schuette, 2020). NifN was not found in the genomes based on the annotation using eggNOG-mapper v2 (Huerta-Cepas *et al.*, 2017; 2019).

**Fig. 2.**
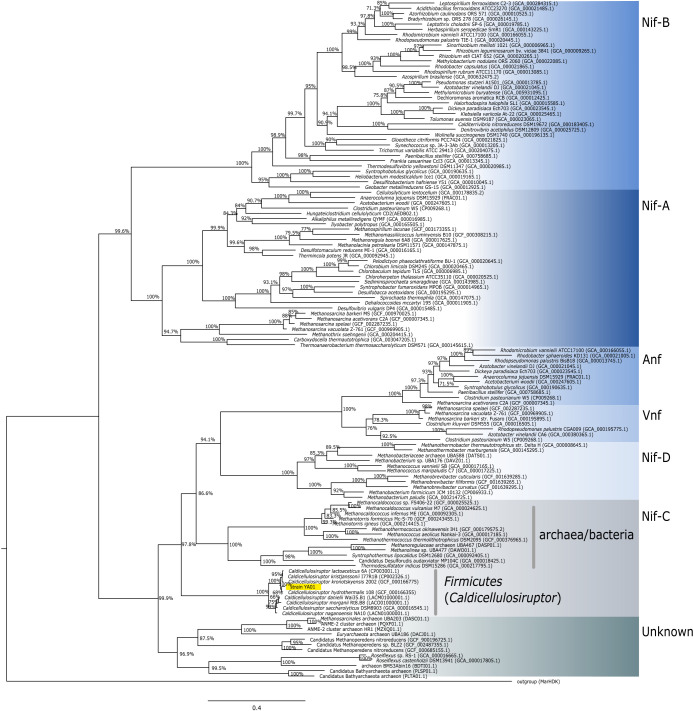
Phylogenetic tree of concatenated NifHDK sequences The phylogenetic tree was constructed by the Maximum Likelihood method with 100 bootstrap replicates. The newly isolated strain, strain YA01 is highlighted in yellow. Among the 276 sequences used, 130 representative taxa are shown. Bootstrap values of more than 50% are indicated at the respective nodes. MarHDK protein sequences in *Rhodospirillum rubrum* ATCC11170 were used as the outgroup. Abbreviations: Nif, Mo-nitrogenase; Anf, Fe-nitrogenase; Vnf, V-nitrogenase; Unknown, uncharacterized nitrogenase homologues. The cluster for Nif (Nif-A, B, C, and D) is shown according to the definition by Poudel *et al.*, 2018.

The amino acid sequences of the nitrogenase structural proteins were similar among the genus (NifH, 98.37±0.41%; NifD, 96.58±1.54%; NifK, 92.05±5.26%). A concatenated NifHDK phylogenetic tree was constructed for all *Caldicellulosiruptor* species possessing nitrogen fixation-related genes (Fig. 2). Strain YA01 clustered with all other members of the genus *Caldicellulosiruptor* within the cluster Nif-C (Fig. 2). The NifHDK sequences of *Caldicellulosiruptor* formed a monophyletic lineage, were placed in the deepest clade in the branch of the Anf/Vinf/Nif-D/Nif-C/Unknown lineage (Boyd and Peters, 2013; Poudel *et al.*, 2018; Garcia *et al.*, 2020), and were distantly related to those in other thermophilic bacteria, such as *Thermoanaerobacterium thermosaccharolyticum* (Nif-A lineage) and *Hydrogenobacter* sp. (Nif-B lineage) (Boyd and Peters, 2013). The thermophilic features of NifHDK did not appear to correlate with the primary structure.

### Growth capability under nitrogen-fixing conditions

**Fig. 3.**
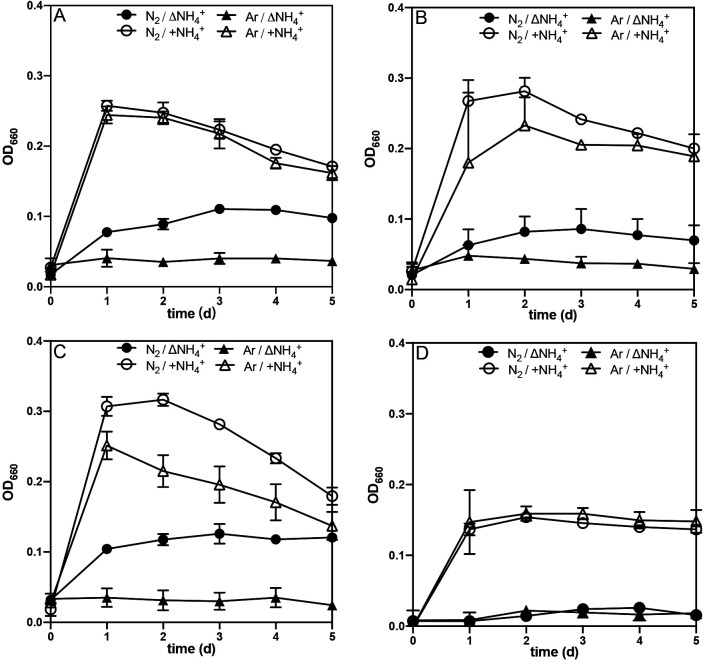
Growth of strain YA01 (A) and its closest species, *Caldicellulosiruptor hydrothermalis* 108 (B), *Caldicellulosiruptor kronotskyensis* 2002 (C), *Caldicellulosiruptor bescii* DSM 6275 (D) with (open symbols) or without (closed symbols) ammonium under a N_2_ or Ar atmosphere. Error bars indicate the standard deviation of three replicates.

Strain YA01 and its three relatives, *C. hydrothermalis* 108, *C. bescii* DSM 6275, and *C. kronotskyensis* 2002, were cultivated in nitrogen-poor medium and ammonium-containing medium under the N_2_ or argon (Ar) gas phase (Fig. 3). *C. bescii*, which did not possess nitrogen-fixing genes, did not grow in nitrogen compound-free medium (Fig. 3D). Strain YA01, *C. hydrothermalis* 108, and *C. kronotskyensis* 2002 showed marked increases in OD in nitrogen compound-free medium under N_2_ gas, but not under the Ar gas phase (Fig. 3A, B, and C). These three strains reached the stationary phase within 2 to 3 days and final OD were 0.05 to 0.14 under N_2_-fixing conditions, corresponding to 1.06×10^7^ to 2.27×10^7^‍ ‍cells‍ ‍mL^–1^. In ammonium-containing medium, the growth of all these strains was faster than in the absence of ammonium and final OD were 0.20 to 0.35. In *C. hydrothermalis* 108 and *C. kronotskyensis* 2002, growth yields in ammonium-containing medium were slightly higher under N_2_ gas than under Ar gas.

### Nitrogenase activity

To test nitrogenase activity, strain YA01 and its relatives *C. hydrothermalis* 108 and *C. kronotskyensis* 2002 were cultivated to the exponential growth phase in nitrogen-poor medium and ammonium-containing medium under the N_2_ gas phase. Acetylene was injected into the vials and incubated at 70°C. The results of ethylene production after a 24-‍h incubation are summarized in Table 1. Ethylene production was observed in all tested strains, even in the presence of ammonium. Strain YA01 showed the highest value among the three strains in the absence of ammonium. The amount of ethylene produced in the presence of ammonium for 24 h was lower than that in its absence in all strains. The suppressive effects of ammonium on *C. kronotskyensis* 2002 were weak (approximately 60% of that in its absence). Acetylene-reducing activities were also observed under an incubation at 78°C for all strains: 9.50±5.05, 8.37±5.53, and 15.0±12.1‍ ‍nmol C_2_H_4_ 10^6^ cells^–1^ 24 h^–1^ by strain YA01, *C. hydrothermalis* 108, and *C. kronotskyensis* 2002, respectively. Activities at 78°C were weaker than those at 70°C.

## Discussion

In the present study, we isolated a bacterial strain by cultivation in nitrogen-poor medium from Nakabusa Hot Spring, Japan. The results of the phylogenetic analysis based on the 16S rRNA gene sequence suggested that this isolate, strain YA01, is a new species in the genus *Caldicellulosiruptor* and is closely related to *C. hydrothermalis*, *C. bescii*, and *C. kronotskyensis*. The results of the genomic analysis indicated that strain YA01 as well as *C. hydrothermalis* 108 and *C. kronotskyensis* 2002 possess a set of nitrogen fixation-related genes (Fig. 1) and their NifHDK formed a monophyletic lineage in the deeply branching group of the NifHDK tree (Fig. 2). Growth capability with N_2_ gas as the sole nitrogen source and acetylene-reducing activity were successfully demonstrated for the new isolate, *C. hydrothermalis* 108, and *C. kronotskyensis* 2002 (Fig. 3 and Table 1). To the best of our knowledge, this is the first study to detect nitrogen-fixing ability in the genus *Caldicellulosiruptor*. The nitrogenase activities of bacteria were previously reported at temperatures up to 70°C by Nishihara *et al.* (2018b) in the chemolithoautotrophic bacteria, *Hydrogenobacter* sp. in the phylum *Aquificae*. The nitrogenase activities of *Caldicellulosiruptor* were detected at temperatures higher than 70°C, *i.e.*, 78°C, which was the maximum growth temperature of strain YA01.

The *nif* gene operons of strain YA01 and its relatives, *C. hydrothermalis* 108 and *C. kronotskyensis* 2002 basically comprised *nifHDKEB* (Fig. 1). Commonly known *nif* gene operons contain the additional gene, *nifN*; however, a homologous gene to *nifN* was not identified in *Caldicellulosiruptor*. *nifN* encodes subunits of the tetrameric protein NifEN (2NifE 2NifN), which is required for the biosynthesis of the iron molybdenum co-factor of Mo-type nitrogenase (Hu *et al.*, 2005, 2006, 2008; Corbett *et al.*, 2006; Burén *et al.*, 2020). Evolutionary studies based on molecular phylogram and comparative analyses of amino acid sequences suggested that *nifN* and *nifK* are paralogous genes that were derived through gene duplication (Raymond *et al.*, 2004; Boyd *et al.*, 2011b). Similar to *Caldicellulosiruptor*, the diazotrophic archaeon, *Methanocaldococcus* sp. FS406-22 also lacks *nifN* (Mehta and Baross, 2006). This finding indicates that the protein coded by *nifK* in these thermophiles performs the same function as NifN. Alternatively, NifE may work without an NifN subunit, as suggested by Garcia *et al.* (2020), because NifE in *Caldicellulosiruptor* showed low similarity with other known NifE (Fig. S2). The phylogenetic trees of NifHDK (Fig. 2) and NifE (Fig. S2) indicated that *Caldicellulosiruptor* has an ancient nitrogen-fixing enzyme system. As proposed in the phylogenetic study of nitrogen fixation-related genes by Garcia *et al.* (2020), Mo-nitrogenase in the genus *Caldicellulosiruptor* may have emerged earlier and then evolved into modern nitrogenases in wide lineages of prokaryotes.

The nitrogenase activities of the *Caldicellulosiruptor* strains were not completely suppressed by the addition of ammonium (Table 1); however, the inhibition of nitrogenase activity by ammonium has been traditionally reported in most diazotrophic bacteria (Dixon and Kahn, 2004). In cellulolytic fermentative diazotrophic bacteria, *Clostridium* sp. in *Firmicutes*, acetylene-reducing activity decreased under the detection limit when ammonium was added (Bogdahn and Kleiner, 1986a, 1986b). However, this activity was not suppressed by ammonium for the thermophilic relative, *Clostridium thermocellum* (now *Hungateiclostridium thermocellum*) (Bogdahn and Kleiner, 1986a; Tindall, 2019). Although the protein, NifA has been shown to regulate the expression of *nif* genes in nitrogen-fixing aerobes in *Proteobacteria* (Merrick, 1992), most anaerobic diazotrophs, including *Caldicellulosiruptor* and *Clostridium*, do not possess the *nifA* gene (Boyd *et al.*, 2015). The evolution of Nif regulation systems from anaerobic to aerobic metabolism is still debatable (Boyd *et al.*, 2015). Further transcriptional and enzymological studies are required to elucidate responses to ammonium in these thermophilic diazotrophs.

*Caldicellulosiruptor* are frequently detected from microbial mats in geothermal springs (Lee *et al.*, 2018; Blumer-Schuette, 2020). Microbial mats are stratified communities of microorganisms with thicknesses of 3 to 5‍ ‍mm and thermophilic microbial mats have been utilized as a model microbial community to investigate the development and maintenance of ecosystems (Taffs *et al.*, 2009; Klatt *et al.*, 2013; Kim *et al.*, 2015; Lindemann *et al.*, 2016; Bernstein *et al.*, 2017; Haruta, 2020). Previous studies focused on primary production in communities in hot spring streams and reported a spatial and temporal distribution and the co-occurrence of carbon-fixing metabolism via oxygenic and anoxygenic photosynthesis, aerobic chemosynthesis (*e.g*., H_2_ and sulfide oxidation), and anaerobic chemosynthesis (*e.g*., sulfur disproportionation) (Rothschild and Mancinelli, 1990; Kimura *et al.*, 2010; Kojima *et al.*, 2016; Tamazawa *et al.*, 2016; Sharrar *et al.*, 2017; Gutiérrez-Preciado *et al.*, 2018; Kawai *et al.*, 2019). Dinitrogen fixation is also required for community development in spring waters that are poor in nitrogen compounds (Kato *et al.*, 2004; Steunou *et al.*, 2006; Kimura *et al.*, 2010; Hamilton *et al.*, 2011; Loiacono *et al.*, 2012). However, possible thermophilic diazotrophs at temperatures higher than 70°C in terrestrial springs have not been clarified. The present results provide important insights into the development of micro-ecosystems in thermal environments. *Caldicellulosiruptor* may utilize organic compounds derived from primary producers at the anoxic layer of microbial mats and provide ammonium to the communities. *Caldicellulosiruptor* possessing the ancient type of nitrogenase may play important roles in carbon and nitrogen cycles not only in modern thermal springs, but also in the early Earth.

## Citation

Chen, Y., Nishihara, A., and Haruta, S. (2021) Nitrogen-fixing Ability and Nitrogen Fixation-related Genes of Thermophilic Fermentative Bacteria in the Genus *Caldicellulosiruptor*. *Microbes Environ ***36**: ME21018.

https://doi.org/10.1264/jsme2.ME21018

## Supplementary Material

Supplementary Material

## Figures and Tables

**Table 1.** Acetylene-reducing activities of strain YA01 and its closest relatives, *Caldicellulosiruptor hydrothermalis* 108 and *Caldicellulosiruptor kronotskyensis* 2002 in the absence and presence of ammonium at 70°C.

Strains		YA01			*C. hydrothermalis* 108			*C. kronotskyensis* 2002	
Conditions*		ΔNH_4_^+^	+NH_4_^+^		ΔNH_4_^+^	+NH_4_^+^		ΔNH_4_^+^	+NH_4_^+^
Acetylene-reducing activity (nmol C_2_H_4_ 10^6^ cells^–1^ 24 h^–1^)**		610±246	18.8±14.3		107±18.3	0.257±0.0694		363±74.4	216±156

*, ΔNH_4_^+^, modified Winogradsky’s nitrogen-poor mineral medium; +NH_4_^+^, ammonium-containing medium (2‍ ‍mmol L^–1^ of NH_4_Cl). **, values were obtained from three culture vials and shown with standard deviations.
