# Supplementary material for: Nitrogen-fixing Ability and Nitrogen Fixation-related Genes of Thermophilic Fermentative Bacteria in the Genus Caldicellulosiruptor
Source: Microbes Environ. 2021 Jun 10;36(2):ME21018. doi: 10.1264/jsme2.ME21018 (PMC8209448; doi:10.1264/jsme2.ME21018)
Supplement: Supplementary file 1 — Supplementary Material [file 36_21018_s1.pdf]

Supplementary Materials for

Title

Nitrogen-fixing ability and nitrogen fixation related genes of thermophilic fermentative bacteria in the genus *Caldicellulosiruptor*

Authors

YUXIN CHEN, ARISA NISHIHARA, and SHIN HARUTA

Corresponding author

SHIN HARUTA

Department of Biological Sciences, Tokyo Metropolitan University, 1-1 Minami-Osawa, Hachioji, Tokyo 192–0397, Japan

E-mail: sharuta@tmu.ac.jp; Tel: +81-42-677-2580; Fax: +81-42-677-2559

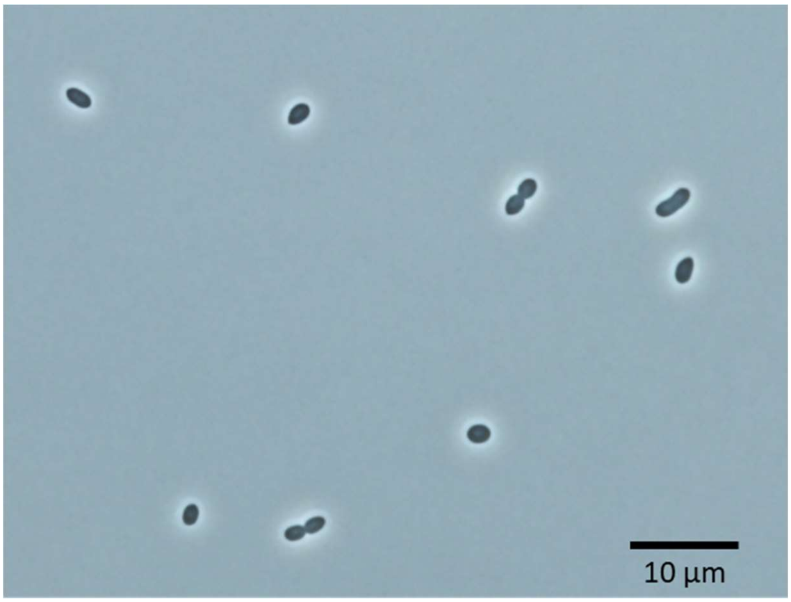

Fig. S1. Phase-contrast microscopic image of strain YA01

The image was captured with optical microscope (Axio Imager A2, Carl Zeiss) equipped with a DP73 camera (Olympus) and CellSens standard software (Olympus). Bar, 10 µm.

Materials and Methods

Proteins that showed high similarity with NifE [≥30% similarity to the NifE either in *Clostridium pasteurianum* (UniProt P10996), *Azotobacter vinelandii* (UniProt P08293), or *Methanococcus maripaludis* (UniProt P0CW55)] were searched for through blastp (v.2.9.0+) (1) in the 10 ORFs upstream and downstream of the ORF for the Nif/Anf/VnfD proteins, that had been used for the Nif/Anf/VnfHDK concatenated tree (Fig. 2). In result, 82 ORFs with length of 400-560 aa were selected. A phylogenetic tree was constructed using the 82 NifE sequences and 144 Nif/Anf/VnfD sequences as used in Fig. 2. The amino acid sequences were aligned using Mafft v7.427 (2). Gapped regions in the alignment were removed with trimAl v1.4 with the parameters (-gt 0.8) (3). Maximum Likelihood tree were constructed using RAxML-NG v. 0.9.0 with LG+F+G4 model with 100 bootstrap replicates (4). Bootstrap support values were recalculated by BOOSTER (v0.1.2) (5). The MarD sequence (WP_011388552.1) were used as the outgroup (6).

References

1. Camacho, C., G. Coulouris, V. Avagyan, N. Ma, J. Papadopoulos, K. Bealer, and T. L. Madden. (2009) BLAST+: architecture and applications. *BMC Bioinformatics* **10**: 421.
2. Katoh, K., K. Misawa, K.-I. Kuma, and T. Miyata. (2002) MAFFT: a novel method for rapid multiple sequence alignment based on fast fourier transform. *Nucleic Acids Research* **30**: 3059–3066.
3. Salvador, C.-G., J. M. Silla-Martínez, and T. Gabaldón. (2009) trimAl: a tool for automated alignment trimming in large-scale phylogenetic analyses. *Bioinformatics* **25**: 1972–1973.
4. Kozlov, A. M., D. Darriba, T. Flouri, B. Morel, and A. Stamatakis. (2019) RAxML-NG: a fast, scalable and user-friendly tool for maximum likelihood phylogenetic inference. *Bioinformatics* **35**:4453–4455.
5. Lemoine, F., J.-B. Domelevo-Entfellner, E. Wilkinson, D. Correia, M. Davila Felipe, T. De Oliveira, and O. Gascuel. (2018) Renewing Felsenstein's phylogenetic bootstrap in the era of big data. *Nature* **556**:452-456.
6. North, J. A., A. B. Narrowe, W. Xiong, K. M. Byerly, G. Zhao, S. J. Young, S. Murali, J. A. Wildenthal, W. R. Cannon, K. C. Wrighton, R. L. Hettich, and F. Robert Tabita. (2020) A nitrogenase-like enzyme system catalyzes methionine, ethylene, and methane biogenesis. *Science* **369**:1094–1098.
7. Poudel, S., D. R. Colman, K. R. Fixen, R. N. Ledbetter, Y. Zheng, N. Pence, L. C. Seefeldt, J. W. Peters, C. S. Harwood, and E. S. Boyd. (2018) Electron transfer to nitrogenase in different genomic and metabolic backgrounds. *Journal of Bacteriology* **200**:1–19.

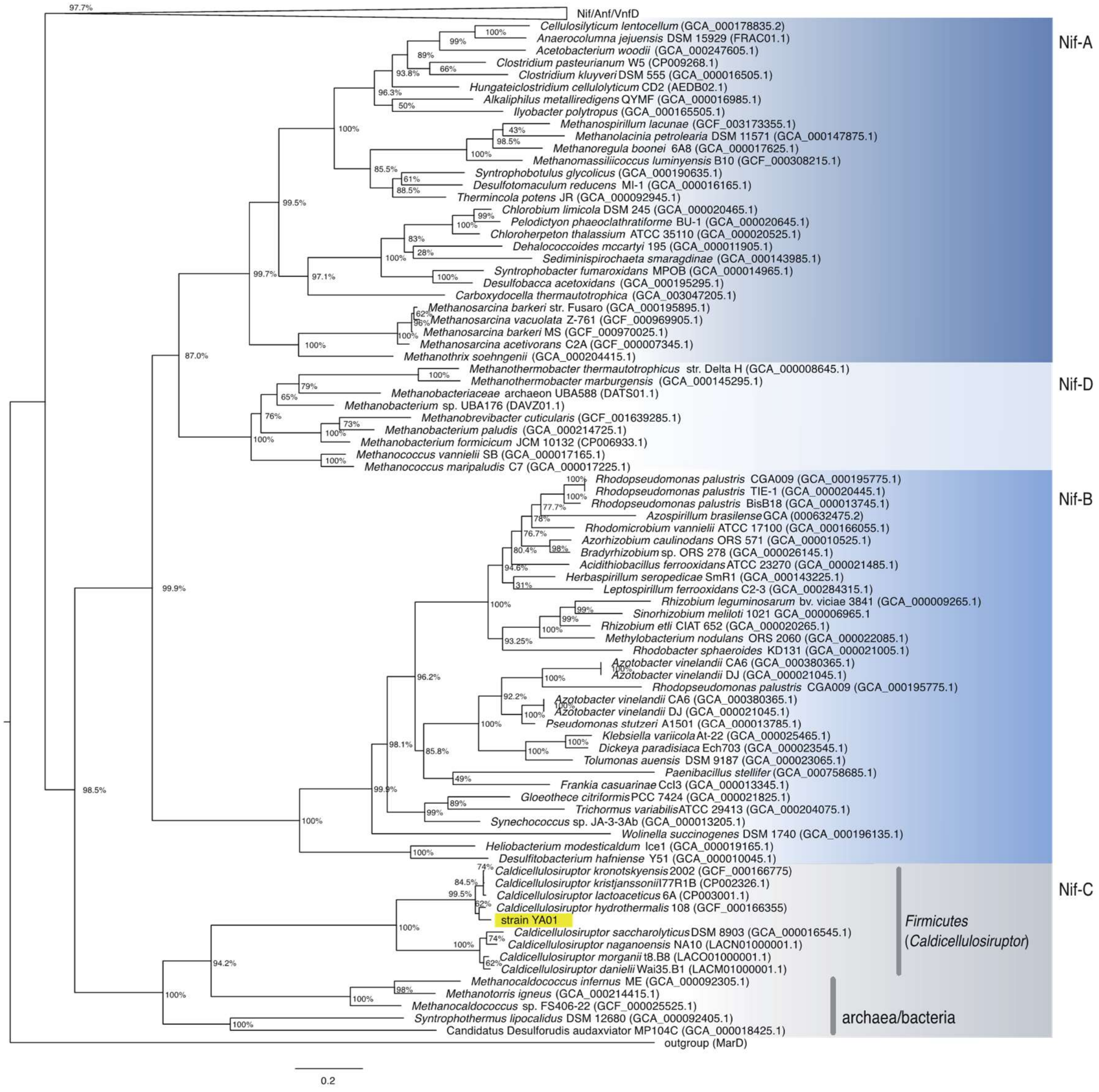

Fig. S2. Phylogenetic tree based on NifE and Nif/Anf/VnfD

The tree was constructed by the Maximum Likelihood method with 100 bootstrap replicates (n=226). The isolated strain, Strain YA01 is highlighted by yellow. The MarD protein from *Rhodospirillum rubrum* ATCC11170 was used as the outgroup. Abbreviations: Nif, Mo-nitrogenase; Anf, Fe-nitrogenase; Vnf, V-nitrogenase. The cluster for Nif (Nif-A, B, C, and D) is shown according to the definition by Poudel *et al*., 2018 (7).
